# The Impact of Interactive Video Games Training on the Quality of Life of Children Treated for Leukemia

**DOI:** 10.3390/cancers16213599

**Published:** 2024-10-25

**Authors:** Aleksandra Kowaluk, Iwona Malicka, Krzysztof Kałwak, Marek Woźniewski

**Affiliations:** 1 Department of Physiotherapy, Wroclaw University of Health and Sport Sciences, 51-612 Wroclaw, Poland; iwona.malicka@awf.wroc.pl (I.M.); marek.wozniewski@awf.wroc.pl (M.W.); 2 Supraregional Center of Paediatric Oncology “Cape of Hope”, Wroclaw University Clinical Hospital, 50-556 Wroclaw, Poland; 3 Department of Pediatric Hematology, Oncology and BMT, Wroclaw Medical University, 50-556 Wroclaw, Poland; krzysztof.kalwak@gmail.com

**Keywords:** children, cancer, physical activity, quality of life, interactive video game

## Abstract

Objectives: The study aimed to assess the impact of interactive video games (IVGs) as a form of physical activity (PA) on the quality of life. Methods: The study used a quality-of-life questionnaire (KIDSCREEN-10) and the HBSC questionnaire. In order to determine individual IVGs training parameters, an initial assessment of cardiorespiratory fitness level was performed, using the Cardio Pulmonary Exercise Test—Godfrey’s progressive protocol. Children in the intervention group participated in 12 interval training sessions using IVGs (Microsoft’s Xbox 360 S console with Kinect,). Results: The study included 21 patients (7–13 years old; 12 boys and 9 girls) treated for acute lymphoblastic leukemia (*n* = 13) and acute myeloid leukemia (*n* = 8). Before the IVGs, all children had insufficient PA levels (90% of children in the intervention group and 90.91% of children in the control group did not engage in any PA during the last 7 days). After the intervention, 80% of the children in the IVGs group undertook PA lasting at least 60 min a day, three times a week. They exhibited better well-being, a subjective feeling of improved physical fitness (*p* < 0.0001), a greater subjective sense of strength and energy (*p* < 0.0001), and less feeling of sadness (*p* = 0.0016) than the children from the control group (*p* = 0.0205). Conclusions: The results of our study confirmed that an attractive form of virtual game or sport is willingly undertaken by children undergoing cancer treatment, and has significant benefits in improving the quality-of-life parameters. There is a clear need to create specific recommendations and rehabilitation models for children with cancer.

## Simple Summary

Despite the continuous increase in the effectiveness of treatment in pediatric oncology and the growing percentage of cured patients, the adverse effects of anticancer treatment remain a serious problem. As a result, the overall quality of life of children with cancer, which is already low, significantly worsens. Social isolation and the prevalence of a sedentary lifestyle intensify the phenomenon of cancer-related fatigue. Virtual forms of physical activity (PA) in the form of interactive video games (IVGs) are forms of PA that are attractive to children, especially in conditions of isolation. Children treated for leukemia who were more physically active during hospitalization showed improvements in parameters such as children’s subjective feelings about their psychophysical health, relationships with family, autonomy, and feelings about the school environment compared to children who did not have access to IVGs. This indicates the need to supplement standard treatment protocols for children with cancer with interactive PA programs.

## 1. Introduction

Despite the continuous increase in the effectiveness of treatment in pediatric oncology and the growing percentage of cured patients, the adverse effects of anticancer treatment remain a serious problem. Anticancer therapy is very aggressive and aimed at rapidly destroying a large number of tumor cells. The occurring episodes of neutropenia [1,2], frequent life-threatening infections [2], as well as painful and unpleasant therapeutic and diagnostic procedures, reduce the comfort of the daily functioning of the treated child [3].

The combination of many unfavorable factors (i.e., high doses of chemotherapeutic drugs, surgical treatment, and radiation therapy) significantly reduces the quality of life of children and adolescents treated for cancer [4]. Bone marrow transplantation, which is sometimes an additional stage of treatment, prolongs a child’s treatment and hospitalization. This stage of treatment is also associated with the risk of adverse sequelae. A common complication of bone marrow transplantation, occurring in about 30 to 70% of cases, is chronic graft-versus-host disease (GVHD) [5,6]. GVHD is a major cause of impaired physical performance and social and psychological impairment [7]. As a result, the overall quality of life of children with cancer, which is already low (compared to healthy peers), significantly worsens [8].

In addition, psychosocial and psychophysical factors (i.e., social isolation, being in an adult-only environment, and the prevalence of a sedentary lifestyle) intensify the phenomenon of cancer-related fatigue (CRF) and have an adverse impact on the level of overall quality of life of treated children [9]. CRF symptoms such as physical exhaustion, sleep disturbances, emotional distress, and cognitive dysfunction very often result in a child’s lack of desire to engage in additional, unforced physical activity [10]. As a consequence of prolonged periods of inactivity and a lack of sufficient levels of physical activity (recommended/necessary during childhood), existing CRF symptoms intensify and physical impairment progresses [11].

Many studies have been conducted that confirm the beneficial effects of physical activity (PA) on the body and mental state of children with cancer. These studies have shown that children who are physically active during the treatment process have better levels of cardiorespiratory fitness, higher muscle strength and flexibility, better functional mobility, and bone mineral density. Improvements in psychological parameters and an improved mood and sleep quality of treated children have also been observed [12,13,14,15]. However, nowadays, children prefer to spend time in front of a screen, and traditional forms of sports are not always as attractive to them as modern technology. Virtual forms of physical effort that are synchronized with images and sound on the screen in the form of interactive video games (IVGs) are forms of physical activity that children are more likely to choose. This has been observed both in the group of healthy children [16] and those with cancer [17,18,19,20]. To date, IVGs used to increase the level of physical activity have been mainly applied to a group of healthy children and young adults [21,22]. Although interactive games are not always an effective solution to increase the physical activity level of healthy children [23], they have shown higher effectiveness in a group of sick children [24,25]. IVGs training has a relatively big advantage over other forms of traditional rehabilitation. First of all, it provides a form of entertainment for children despite the fact that IVGs require a great deal of physical effort on the part of the sick child and is actually a form of training. Such training resembles a game for the treated children, as there are elements of competition and motivational elements in the form of sounds (ovations, applause) [26]. In addition, IVGs training can be carried out in the patient’s hospital room. This is very important due to the frequent need to isolate oncological patients and the high risk of infection [27]. Hospitalized children have limited opportunities to enjoy commonly available forms of physical activity, and IVGs give them this opportunity in a virtual form in a hospital setting. IVGs training allows individual selection of the type of game according to the child’s interests. They also allow for gradual effort intensity adjustments. It is a convenient form of training because it does not require meeting sophisticated conditions, nor does it require special staff training. Such IVGs training with individually selected intensity parameters determined by the initial cardiorespiratory fitness test is safe and can even become part of the rehabilitation program for children who are undergoing intensive cancer treatment [28,29].

The disadvantages of training with IVGs are the lack of contact with a larger group of children and the inability to create their own spontaneous physical activity. Also, this form of physical activity is still time spent in front of a monitor screen [30]. In addition, the results of other researchers have shown that the level of interest in IVGs decreases with the time of participation in the game. Such a phenomenon is not observed as often in traditional forms of physical activity. When the interactive game is no longer new, children lose interest and their activity level decreases [31].

IVGs may represent a novel method of rehabilitation for children undergoing treatment for neoplasms. However, they are not yet widely used in hospitals, where traditional forms of improvement and rehabilitation prevail. Usually, no forms of physical play are proposed to children during hospitalization. Training in the form of video games attractive to children can significantly improve parameters affecting the perceived quality of life of sick children. Such forms of recreation or sport are an effective way to increase the level of daily physical activity for children undergoing cancer treatment. However, there is a lack of research that would determine the impact of specific parameters of IVGs (intensity, game time, frequency of training) on the level of quality of life of children with cancer.

Interactive forms of physical activity, which are becoming an increasingly popular method of rehabilitation, effectively increase the level of physical activity of treated children and, through this, have a positive impact on the quality-of-life parameters. The purpose of this study was to assess the impact of interactive video games training (IVGs), as a form of physical activity, on the level of quality of life of children treated for leukemia.

## 2. Study Design

### 2.1. Participants and Recruitment

The study included a group of children who were patients of the Department of Bone Marrow Transplantation, Oncology and Pediatric Hematology of the Jan Mikulicz-Radecki University Clinical Hospital in Wroclaw. The patients underwent oncological treatment (chemotherapy cycles in the hospital conditions). The duration of treatment did not exceed 6 months from the moment of cancer diagnosis. The study included 21 school-aged children [29] (7–13 years old; 12 boys and 9 girls) (Table 1) treated for acute lymphoblastic leukemia (ALL) (*n* = 13) and acute myeloid leukemia (AML) (*n* = 8). The subjects were enrolled using a computer program that randomly selected children for the study. Children were also randomly assigned to the intervention and control groups. Subjects in the intervention group participated in an in-hospital rehabilitation program using interactive video games. Children in the control group were not included in any rehabilitation program and did not report having any interactive video game set. Dividing the recruited children into an intervention group and a control group allowed us to test whether participation in the IVGs program affected parameters describing the quality of life of children undergoing cancer treatment. In addition, it was possible to assess whether IVGs would be an attractive form of physical activity for children that would reduce their sedentary behaviors.

Body height and weight were measured for each participant before the study began. Each participant was diagnosed with cancer based on a series of detailed examinations, including cytologic evaluation, immunophenotype, and genetic and molecular evaluation. Patients treated for ALL were included in the international protocol for the treatment of children and adolescents with acute lymphoblastic leukemia (AIEOP-BFM ALL 2017 protocol) [32], while patients treated for AML were included in the international protocol for the treatment of children with acute myeloid leukemia (AML-BFM 2012 protocol) [33]. All children were classified into three risk groups (SR—standard-risk group; IR—intermediate-risk group; HR—high-risk group) based on the following criteria: leukocyte count, patient age, cytogenetic findings, achievement of remission, leukemia type, and response rate to treatment. There were no statistically significant differences between the risk groups.

Inclusion and exclusion criteria were defined. Inclusion criteria included diagnosis of malignant neoplasm (ALL or AML), age between 7 and 13 years, inpatient treatment in a hospital ward, hospital stay >7 days, applied chemotherapy, no physical disability, no comorbidities, independent arrival for the examination, and consent of parent/legal guardian for participation in the study. Exclusion criteria included hemoglobin <8 g/dL, platelet count <20,000/mm^3^, infectious disease, elevated body temperature >38 °C, intellectual disability of the child, psychiatric conditions, and eating disorders.

### 2.2. Children from the Intervention Group

The group included 10 patients: (5 boys, 5 girls). Children aged 7–13 years (mean age 11.3 years, SD 2.0 years) were treated for ALL (*n* = 8) and AML (*n* = 2). The mean body height of the children in the intervention group was 149.1 cm, SD 13.76 cm, and the mean body weight was 46.59 kg, SD 16.0 kg (Table 1). Children in the intervention group were included in a rehabilitation program of 12 sessions of IVGs (Microsoft’s Xbox 360 S console with Kinect) during the hospitalization period.

### 2.3. Parents of Children from the Intervention Group

Parents of children in the intervention group (mean age 39 years, SD 3.3 years) had vocational education in 60% of the cases. Secondary education was declared by 30% of parents, while 10% of parents had higher education. The occupational situation of parents of treated children was characterized in most cases by not taking up gainful employment during their child’s hospitalization. As many as 90% of parents declared that during the period of their child’s cancer and active treatment process, their only source of income was a care allowance for caring for their sick child. Taking up gainful employment and professional activities was shown by only 10% of parents of children from the intervention group. The marital status of parents of children in the intervention group indicated that 60% of these people were in an informal relationship with a partner, and had children from a previous relationship. The remaining 40% of those staying with children at the clinic were married mothers with children. Rural areas were declared as the place of residence by 60% of the parents, while the remaining 40% of the families were settled in cities (10% of the parents declared a city of up to 50,000 inhabitants; 20% of the parents declared a city of 50,000 to 100,000 inhabitants; 10% of the parents declared a city of more than 300,000 inhabitants). The material situation of 20% of families indicated a very good condition, 60% of families declared an average material condition of the family, and 20% declared a rather bad material condition of the family.

### 2.4. Children from the Control Group

The group of children included 11 patients (7 boys, 4 girls). Patients aged 7–13 years (mean age, 10.08 years, SD 1.9 years) were treated for ALL (*n* = 5) and AML (*n* = 6). The mean body height of children in the control group was 140.0 cm, SD 16.5 cm; the mean body weight was 36.45 kg, SD 9.9 kg) (Table 1). Children in the control group did not participate in any rehabilitation intervention.

### 2.5. Parents of Children from the Control Group

Of the parents of the children in the control group (mean age 40.9 years, SD 2.6 years), 45.4% had a vocational education, 36.4% had a secondary education, while 18.2% had a higher education. The employment situation of 81.9% of parents of the children in the control group was characterized by the absence of gainful employment during their child’s hospitalization. The only income of a parent staying with their child for treatment at the clinic was a care allowance for caring for their sick child. The rest of the parents (18.1% of parents) were economically active and took up gainful employment. Of the parents of the treated children in the control group, 54.5% were in an informal relationship with a partner and had children from a previous relationship. Mothers who were married with children accounted for 27.3% of the group, while 18.2% were married fathers with children. Rural areas were reported as the place of residence by 18.2% of parents, 45.4% of parents resided in cities of up to 50,000 inhabitants, 18.2% of parents resided in cities of 50,000 to 100,000 inhabitants, and 18.2% resided in cities of more than 300,000 inhabitants. In the control group, 18.2% of families had a very good financial situation, 63.6% of families had an average financial situation, and 18.2% of parents declared a rather bad financial situation of the family.

## 3. Research Methods

### 3.1. Quality of Life

The quality of life of the children studied was assessed using the KIDSCREEN-10 questionnaire (shortened version). The short version of the questionnaire evaluates the health-related quality of life of children and adolescents. The following aspects were assessed: children’s subjective feelings related to their physical and mental health, relationships with parents and peers, children’s autonomy, and feelings related to the school environment. The questions pertained to the last 7 days. The evaluation was carried out in the intervention and control groups at two time points (at the beginning of the study period and after its completion).

### 3.2. Physical Activity Level

The International Health Behaviour in School-aged Children (HBSC) questionnaire was used to assess the level of physical activity and sedentary behavior in the last 7 days. The MVPA (moderate to vigorous physical activity) index was evaluated. This was the number of days of the week and the number of hours per day during in which children performed physical activity lasting a total of at least 60 min continuously. The frequency of engaging in physical exercises independently, on their own (excluding sports as part of school activities), was also assessed. The questionnaire also evaluated time spent in front of a TV and computer screens. The assessment of children’s physical activity levels was conducted in both the intervention and control groups at the beginning of the research period and immediately after its completion.

### 3.3. Level of Cardiorespiratory Fitness

To select individual IVGs training parameters for each patient, an initial assessment of the cardiorespiratory fitness level was conducted at the beginning of the study using the CPET (Cardio Pulmonary Exercise Test). The test was carried out according to the principles of Godfrey’s progressive protocol [34,35].

The results of the CPET test were the basis for planning the individual intensity of IVGs training for children in the intervention group. Each subject began the test with a 3-min warm-up (warm-up power of 15 W for children with a body height of 120–150 cm; power of 20 W for children with a body height >150 cm). The warm-up was followed by the actual test, in which the load was increased in 1-min intervals by 15 or 20 watts (depending on the patient’s body height). The test interruption criteria were defined, which at the same time formed the basis for determining peak effort. These were HR_peak_ > 180 beats per minute; respiratory exchange ratio (RER_peak_) value > 1.0; and a decrease in pedaling frequency below 60 revolutions per minute, despite strong verbal encouragement from the investigator.

Respiratory parameters during the CPET test were recorded with a portable COSMED K4b2 ergospirometry system. The test load at scheduled intervals was imposed by an ASPEL CRG200 bicycle ergometer, which was compatible with the CardioTEST exercise testing system and the AsTER cardiac rehabilitation system.

The peak oxygen uptake (VO_2peak_) was calculated as the average value of the last 30 s of the exercise test [36]. Children were not subjected to maximal exercise and maximal oxygen uptake (VO_2max_) was not assessed, only the peak value (VO_2peak_). The test evaluated the peak values of the parameters tested due to patient safety and the existing contraindications for undertaking maximum efforts in the group of patients with cancer undergoing intensive treatment [29,37].

## 4. Interactive Video Game Training in the Intervention Group

The IVGs sessions, which were attended by children in the intervention group, consisted of participation in 12 interval training sessions using interactive video games. The kit for the IVGs consisted of a console with a motion sensor (Xbox 360 console, Microsoft) and a screen displaying the game image. The children participated in the trainings for four consecutive weeks, with sessions held at a frequency of three times a week. Before starting the actual training sessions, each participant took part in an introductory session. The introductory session lasted 3 min and consisted of taking up an activity in any game to learn how the console works.

**Figure 1:**
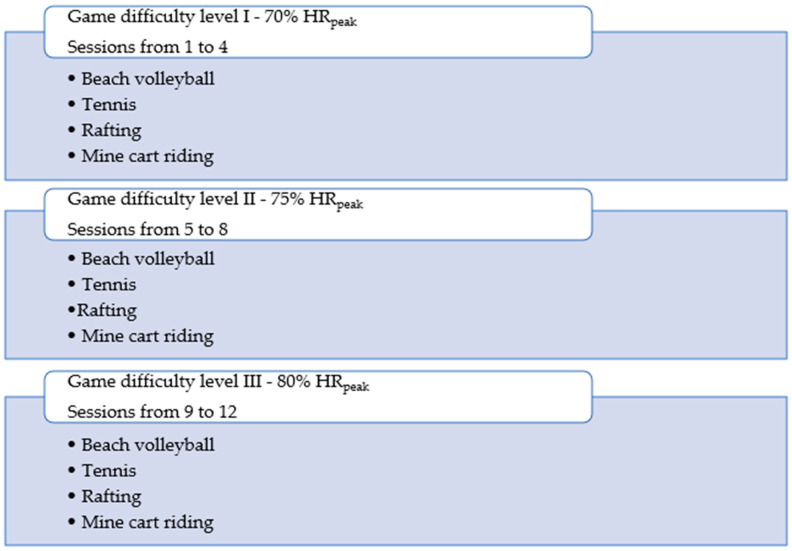
Intervention scheme of interactive video games selected games [29].

Each actual training session consisted of four different interactive games: Kinect Sports (beach volleyball), Kinect Sports Season Two (tennis), and Kinect Adventures (rafting, mine cart riding). Each game lasted 5 min. Between each game, the participant rested for 1 min (Figure 1). The type of physical effort during the selected games was most similar to continuous physical effort, while the 1-min breaks made the assigned training an interval training. The intensity of the training was selected individually for each participant in the study based on the results of the initial CPET test. The individually programmed training was characterized by a moderate level of intensity and was based on the relationship between oxygen uptake and heart rate in children [38]. In order to gradually increase the intensity of the training, three consecutive levels of game difficulty were introduced (sessions from 1 to 4—game difficulty level I—70% HR_peak_; sessions from 5 to 8—game difficulty level II—75% HR_peak_; sessions from 9 to 12 difficulty level III—80% HR_peak_) (Figure 1). The target HR values for each participant in each successive game difficulty interval were calculated based on the HR_peak_ value achieved in the CPET test. The gradual introduction of more advanced levels of play was aimed at each player achieving the target HR values for each session. Control of HR parameters and ongoing evaluation of exercise intensity was performed in real time using a physical activity monitor (Polar M 430) and the Polar Flow app compatible with the PA monitor. Each child monitored his or her HR parameters. Verbal motivation from the researcher and parents was essential for the children to achieve the set HR values for the session [29,38].

## 5. Ethics

This study received a positive opinion from the Senate Research Ethics Committee, Wroclaw University of Health and Sport Sciences. Consent number: 7/2018; Date of approval: 8 March 2018.

## 6. Statistical Analysis

The statistical analysis was performed using GraphPad Prism 7 software (Institute of Immunology and Experimental Therapy, Wroclaw, Poland). The normality of the data distribution was assessed using the Shapiro–Wilk test. Parameters defining the characteristics of the studied groups were presented by providing descriptive statistics (arithmetic mean, standard deviation). To assess the statistical significance of the differences in the results characterizing the studied groups, the Student’s *t*-test for independent groups with Welch’s correction was used when the evaluated data had a normal distribution. The Mann–Whitney U test was used to assess the statistical significance of the differences in the results describing the study groups when the data evaluated had a distribution different from normal. The Kruskal–Wallis test was used for global analysis of differences between groups, followed by the Dunn’s test to assess the significance of differences between selected variables. Spearman’s rank correlation was used to evaluate the relationship between the level of physical activity and parameters assessing the level of quality of life of children in each group. The significance level was taken as *p* < 0.05.

## 7. Results

Before the training intervention with IVGs, there were no statistically significant differences in physical activity levels between the intervention group and the control group. This was evident in the assessment of the number of days per week during which children engaged in physical activity for at least 60 min per day (MVPA index)—HBSC 1 (*p* = 0.99) (Table 2) and in the frequency of high-intensity physical activity—HBSC 2 (*p* = 0.99) (Table 2).

After the training intervention with IVGs, a statistically significant difference was observed in the level of physical activity between the intervention and control groups (HBSC 1 and HBSC 2 questions; *p* = 0.0048 and *p* = 0.0003, respectively) (Table 2).

Children who were physically active during the leukemia treatment process had a better sense of well-being and a subjective feeling of higher physical fitness (*p* < 0.0001). Children receiving IVGs training showed a greater subjective sense of strength and energy (*p* < 0.0001), less feeling of sadness and apathy (*p* = 0.0016), and did not feel loneliness as much as children in the control group (*p* = 0.0205) (Table 3). Children in the intervention group who spent time actively significantly felt satisfaction with being able to engage in activities that they enjoyed (*p* = 0.01) (Table 3).

The percentage distribution of HBSC survey responses showed that children in the intervention and control groups did not engage in physical activity lasting at least 60 min a day before the study period. As many as 90% of the children in the intervention group and 90.91% of the children in the control group reported that they had not engaged in such physical activity in the past 7 days. All of the children in both the intervention and control groups declared that they engaged in significant physical efforts less than once a month or never. Detailed analyses are provided in Table S1 in the Supplementary Materials. The survey responses and questions were included in a previous paper [39].

Children in the intervention group were significantly more likely to engage in physical efforts on a weekly basis after receiving a training program using IVGs. Physical activity with a frequency of three times a week was declared by 80% of the children in the IVGs group. The remainder of the IVGs program participants (20% of the children) engaged in physical activity twice a week for at least 60 min a day in total. Children in the control group did not increase their level of physical activity. Detailed analyses are provided in Table S1 in the Supplementary Materials.

Before the start of the training period, both children in the intervention group and the control group declared a lack of well-being and an insufficient level of physical fitness (90% and 90.91% of children, respectively). Before starting the training, all children in both groups reported frequent feelings of fatigue and a lack of energy. Children’s declarations included responses such as “I was never full of energy” or “I was rarely full of energy.” Children in the intervention and control groups felt sadness very often (60% and 63.64% of children, respectively) or always (40% and 36.36% of children, respectively) before the start of the study period. Children in both groups experienced loneliness before the IVGs intervention. Participants of the IVGs program at the beginning of the study period felt lonely quite often (60% of children), very often (30% of children), and always (10% of children). Children in the control group felt lonely quite often (27.27% of children) and very often (72.73% of children). Children in the intervention group before the IVGs program declared that they never (10% of children) had time for themselves. In addition, 30% and 60% of children in the intervention group responded that they rarely or quite often had free time and could do what they wanted. Correspondingly, children in the control group answered 36.36% of the same question that they rarely or quite often (63.64% of children) had enough time for themselves. Detailed analyses are provided in Table S2 in the Supplementary Materials. The survey responses and questions were included in a previous paper [39].

Also prior to the study period, all children in both the intervention and control groups had good relationships with their parents: very often (10% and 18.18%, respectively) and always (90% and 81.82%, respectively). As many as 70% of the children in the intervention group and 81.82% of the children in the control group responded that they had not engaged in joint play with peers in the last 7 days. All children in both groups declared difficulties at school and problems with concentration and the ability to focus attention in the preintervention period. Detailed analyses are provided in Table S2 in the Supplementary Materials.

After the training intervention, the children in the IVGs group declared noticeable improvements in subjective feelings related to their physical and mental health. These children responded that they felt moderately (20% of children) and very (80% of children) good and physically fit. All of the children in the IVGs group felt strong and energetic quite often (30% of the children) and very often (70% of the children) after the training period. It was also observed that children who underwent IVGs training rarely felt sad (80%) and rarely felt lonely (70%). These children also felt that they had more time for themselves and could allocate time to activities that they enjoyed. All of the children declared that they could quite often (40% of the children) and very often (60% of the children) do whatever they wanted in their free time.

Relationships with parents and peers, feelings related to the school environment, as well as the ability to concentrate and pay attention remained at similar levels. Detailed analyses are provided in Table S2 in the Supplementary Materials.

Children who participated in IVGs training and spent less time during the day in a sitting position (reduced sedentary behavior) declared that they felt more strength and energy (higher well-being) (*p* = 0.03; r = −0.76). Detailed analyses are provided in Table S3 in the Supplementary Materials.

Children in the control group who spent less time in front of the monitor screen also exhibited less feelings of loneliness (*p* = 0.02; r = −0.71). It was also observed that children who spent less time in front of the screen on days off had better concentration skills and the ability to focus on the activities performed (*p* = 0.02; r = 0.79). Detailed analyses are provided in Table S4 in the Supplementary Materials.

## 8. Discussion

In our study, we assessed whether an intervention of interactive video games aimed at increasing the level of physical activity influenced the quality-of-life parameters for children undergoing cancer treatment. The results of our study showed that prior to the IVGs intervention, all children had inadequate levels of physical activity. As many as 90% of the children in the intervention group and 90.91% of the children in the control group declared that they had not engaged in any physical activity in the last 7 days.

The results of other researchers also confirm significantly reduced and worrying levels of daily physical activity and an increase in sedentary behavior in the group of children with cancer. Studies show that the population of children diagnosed with cancer does not remain active during and after treatment. A long period of remission of the disease also does not increase physical activity levels [40]. Also, a study by Braam et al. confirms that children with cancer exhibit inadequate levels of PA and do not independently undertake any form of exercise that could improve their fitness. Researchers have shown that these children spend 80% of their entire day in a sedentary position, and their physical effort is only related to performing activities of daily living [13]. Children undergoing cancer treatment do not engage in physical activity for a total of at least 60 min a day and thus do not meet the recommendations for the MVPA index [41]. The MVPA index value recommended by the World Health Organization is aerobic exercise lasting at least 60 min a day for 7 days a week at moderate to vigorous intensity [42].

Therefore, early diagnosis and assessment of the magnitude of the problem and existing psychophysical deficits of children with cancer is important. Psychosocial factors, family involvement, habits, and the future prospects of engaging in physical activity in young and adult life are already important at the stage of intensive cancer treatment. The long-term care of children with cancer and providing them with support, such as through physical activity interventions, can play a key role in the success of the treatment process and the maintenance of full fitness. The improved well-being that PA (physical activity) interventions can provide translates into better well-being for the child, while children’s improper habits acquired during treatment often become entrenched and remain even after the treatment period is over [43].

In our study, we observed that children in the intervention group eagerly took up the training sessions offered to them using interactive video games. The IVGs constituted an attractive form of physical activity for them. Children in the IVGs group had statistically significantly higher weekly PA levels after undergoing the training program. As many as 80% of these children engaged in physical activity lasting at least 60 min a day three times a week. After the IVGs training program, children in the intervention group assessed that participation in interactive games was a physical effort for them, not a form of play. After the intervention, 80% of children from the IVGs group declared that they undertook physical effort at a significant level (i.e., one that caused a feeling of fatigue, shortness of breath, and increased sweating) 2–3 times a week. However, the full attendance of the intervention group participants in all trainings confirmed that although the children perceived the physical effort given to them as significant, they were very willing to participate in it. Sometimes, children even demanded a higher frequency of classes per week because the IVGs were a form of fun and pleasantly spent time for them. This is confirmed by the analysis of the quality-of-life parameters. As many as 80% of the children declared that they felt very good and were physically fit after the training program with IVGs, while before the IVGs program, 90% of the children in the intervention group declared a lack of well-being and physical fitness.

Interactive video games additionally had a positive effect on improving parameters such as relationships with parents and peers, children’s autonomy, and feelings related to the school environment. Children who underwent IVGs training rarely felt sadness (80%) and rarely felt loneliness (70%). These children also felt that they had more time for themselves and could allocate time to activities that they enjoyed. All children declared that they could quite often (40% of the children) and very often (60% of the children) do whatever they wanted in their free time.

The results of other researchers confirm the low quality of life of children treated for cancer. Saleh et al. observed high levels of anxiety among children undergoing hospitalization, as well as frequent feelings of worry and communication problems. The children also reported frequent nausea and cognitive problems. Researchers observed that these factors have a negative impact on the perception of the overall quality of life of treated children [44]. In a study by Bult et al., it was observed that children treated for cancer have a reduced quality of life, which is caused by frequent joint and muscle pain, nausea, changes in taste and smell of consumed foods, worrying, as well as cognitive problems and change in appearance [45].

Li et al. in their study defined six groups of factors that negatively affect the quality of life of children with cancer. These were gastrointestinal symptoms, emotional symptoms, neurological symptoms, mucosal symptoms, changes in self-perception, and somatic symptoms. Researchers have observed that if there is a disturbance within even one group of factors, it will significantly reduce the quality of life of treated children [46].

The deteriorated quality of life in children with cancer, as well as a lowered mood, certainly do not motivate them to engage in physical activity and further lower their already low PA levels [47]. In contrast, in a study by Götte et al., it was observed that children undergoing hospitalization and active cancer treatment are very eager to engage in rehabilitation and exercise. Patients actively try to contribute to faster recovery by systematically participating in an in-hospital rehabilitation exercise program. The main motivation for children with cancer to take up active forms of exercise was the improvement in physical fitness and the better mental well-being that they experienced after taking up the activity [48].

Studies also confirm that physical activity interventions have a beneficial effect on improving the psychophysical state of children undergoing cancer treatment. Improvements are observed in parameters such as a reduction in fatigue, an improvement in joint range of motion, an increase in muscle strength, an improvement in bone mineral density, and an improvement in aerobic capacity and functional mobility [49,50].

The obtained results in terms of improved quality of life for the studied children after the implemented IVGs intervention are probably due to increased musculoskeletal and cardiorespiratory fitness. Higher physical fitness and better levels of cardiorespiratory fitness make children feel less discomfort when performing daily activities, and thus they feel more independent and inclined to undertake greater physical efforts. This causes children to more willingly engage in additional, unforced physical activity that they were previously unable to do.

Sometimes in the course of cancer treatment, it is necessary to isolate patients, and then an interactive form of sport allows the child to transport themselves in imagination and thoughts to a field, court, swimming pool, etc. This certainly has an impact on the patients’ mood and quality-of-life parameters. Often, a virtual sports game provides motivation to take up additional activity during the hospitalization period and to stop sedentary behavior. The results of other researchers have confirmed that even the traditional form of physical exercise (not interactive) is a form of fun for children and is a motivation for them to stop sedentary behavior and take up physical activity [48]. This further confirms the need to introduce varied, novel and attractive forms of physical activity in a group of children with cancer. Such forms of PA should be enjoyable for patients, and should be individually tailored according to interests and individual physical and psychological capabilities [51].

The results of our study confirmed that an attractive form of virtual game or sport is willingly undertaken by children undergoing cancer treatment, and also brings significant benefits in improving the quality-of-life parameters. There is a clear need for specific recommendations and models of rehabilitation for children with cancer. The general recommendations that currently exist are mainly directed at adults undergoing oncological treatment and are not sufficient and appropriate for children. It would be necessary to include rehabilitation procedures also in situations where children require special care during certain periods of different treatment protocols.

## 9. Strength and Limitations of This Study

This study included a small number of patients so further research should be conducted to increase the size of the group. The proposed training was carried out in one clinical center, and the intervention itself concerned a narrowed group of patients because only children diagnosed with leukemia were included in the group. The psychophysical state of the treated children before the cancer diagnosis is also unknown, so the results presented should not be generalized. The treatment protocol may have influenced the results; nevertheless, due to the small size of the groups, no such analysis was performed. To improve the results of our study, it would be good to increase the size of the groups accordingly in the future with reference to the type of treatment.

The socioeconomic status of the families of children treated for cancer was assessed in our study, but no additional correlations were made with factors describing the quality of life of the children studied. In our study, we focused on evaluating the impact of the intervention on the quality of life of hospitalized children. Assessing the impact of family socioeconomic status on the quality of life of children with cancer can certainly reveal interesting correlations but requires separate studies. Our project assumed that in the situation of prolonged stays in the clinic, prolonged social isolation (family, peers), and isolation from the school environment, all participating children had similar social and living conditions. The socioeconomic status of the family probably had little impact on the quality of life of the treated children.

## 10. Future Research Directions

The results of our study demonstrate the necessity to supplement treatment protocols for children with cancer with new, interactive forms of physical activity and rehabilitation. Specific improvement models for this group of patients should be created, which, by increasing the level of physical activity, would improve the quality-of-life parameters. New forms of activity and rehabilitation would prevent the appearance of early and late adverse effects of cancer treatment, including osteoporosis, loss of cardiorespiratory capacity, contractures, and muscular atrophy. Improvements in physical parameters would benefit psychological parameters, thereby enhancing the quality of life of children undergoing cancer treatment.

## 11. Conclusions

Children treated for leukemia who were more physically active during the hospitalization exhibited a better quality of life compared to children who did not have access to interactive games. It was observed that interactive video games positively influenced improvements in parameters such as children’s subjective feelings about their psychophysical health, relationships with immediate family members, children’s autonomy, and feelings related to the school environment. The children felt joy and satisfaction with their successes and achievements after participating in the IVGs exercise. During hospitalization and treatment, children have few opportunities to accomplish something or reach new goals. This is very necessary for the child’s proper development. Participation in IVGs allows them to achieve good results, thanks to which children can feel joy and satisfaction. These feelings significantly improve the quality of life. This indicates the need to supplement standard treatment protocols for children with cancer with interactive physical activity programs. This will have a positive impact on patients’ mood and the quality of their daily functioning. Treatment protocols for children with cancer focus only on anticancer treatment. Paying attention to patients’ emotional state, daily activities, quality of life, and psychophysical state would be a valuable part of treatment. Currently, physical activity is recommended at every stage of cancer treatment, although it remains an underappreciated component of cancer prevention and therapy. Training with IVGs can become an integral part of the in-hospital rehabilitation process. Exercises using IVGs, delivered to the child in the form of play, can contribute to increasing the level of daily physical activity of treated children. In addition, this form of physical activity can provide respite from illness and distract the child from unpleasant medical procedures. This will consequently improve their quality of life. Children with cancer who engage in physical exertion during the treatment process will be able to participate more actively in social and professional life in the future.

The results of our study should encourage physiotherapists and those working with children with cancer to introduce IVGs as a permanent part of the routine rehabilitation process.

## Figures and Tables

**Table 1:** Characteristics of study participants by gender.

	Intervention Group *n* = 10			Control Group *n* = 11			*p*-Values Intervention Group vs. Control Group	
Determinant Variables	Mean ± SD	Boys Mean ± SD *n* = 5	Girls Mean ± SD *n* = 5	Mean ± SD	Boys Mean ± SD *n* = 7	Girls Mean ± SD *n* = 4		
Age (years)	11.3 ± 1.9	11.4 ± 2.2	11.2 ± 2.0	10.08 ± 1.9	10.15 ± 2.0	9.9 ± 2.2	0.38	The Mann-Whitney U test
Height (cm)	149.1 ± 13.76	154 ± 15.36	144.2 ± 11.39	140 ± 16.5	141.7 ± 18.42	137 ± 14.51	0.51	The Mann-Whitney U test
Weight (kg)	46.59 ± 16.01	49.6 ± 17. 8	43.5 ± 15.4	36.45 ± 9.9	36.57 ± 10.86	36.25 ± 9.43	0.24	The Mann-Whitney U test
Treatment duration until start of the intervention (months)	6.4 ± 1.6	6.0 ± 2.0	6.8 ± 1.3	6.3 ± 1.7	6.4 ± 2.1	6.0 ± 1.15	0.87	The Student’s *t*-test with Welch’s correction
HR at rest	86.5 ± 3.4	86.2 ± 4.2	86.8 ± 2.9	84.9 ± 4.16	85.1 ± 4.95	84.5 ± 2.89	0.35	The Student’s *t*-test with Welch’s correction

**Table 2:** Statistical significance of differences in HBSC questionnaire scores between the intervention and control groups before and after the IVGs intervention.

Intervention Group vs. Control Group	The Difference Between the Ranks	*p*-Value Before Intervention	The Difference Between the Ranks	*p*-Value After Intervention
*Dunn’s test*				
**Number of days per week during which the child engaged in physical activity for at least 60 min (MVPA indicator)—HBSC 1**	1.22	0.99	281.0	0.0048 *
**Frequency of engaging in high-intensity physical efforts—HBSC 2**	−0.97	0.99	−361.5	0.0003 *
**Number of hours per week spent watching TV/films (h)—HBSC 3**	44.9	0.65	−123.0	0.22
**Number of hours on days off spent watching TV/movies (h)—HBSC 3.1**	22.7	0.82	−87.53	0.38
**NA ^a^ games per week (h)—HBSC 4**	−26.05	0.79	−127.0	0.20
**NA ^a^ games on days off (h)—HBSC 4.1**	−3.48	0.97	−159.9	0.11
**Use of devices like computer, tablet, smartphone, etc. per week (h)—HBSC 5**	−3.87	0.97	−83.27	0.40
**Use of devices such as computers, tablets, smartphones, etc. on days off (h)—HBSC 5.1**	28.69	0.77	−146.3	0.14

Note: * Results showing statistical significance; and ^a^ NA—non active video games.

**Table 3:** Statistical significance of differences in quality-of-life scores between the intervention group and controls before and after the intervention.

Intervention Group vs. Control Group	The Difference Between the Ranks	*p*-Value Before Intervention	The Difference Between the Ranks	*p*-Value After Intervention
*Dunn’s test*				
**Well-being and physical fitness—KID 1**	−5.11	0.86	124.6	<0.0001 *
**A sense of strength and energy—KID 2**	−10.23	0.72	120.5	<0.0001 *
**Sense of sadness** **—** **KID 3**	1.4	0.96	−89.89	0.0016 *
**Perception of loneliness** **—** **KID 4**	−5.80	0.84	−66.15	0.0205 *
**Having time for oneself—KID 5**	−7.67	0.79	6.63	0.82
**Doing activities one felt like doing—KID 6**	14.21	0.62	69.31	0.01 *
**Good relations with parents—KID 7**	3.15	0.91	3.48	0.90
**Good relations with peers, fun—KID 8**	11.29	0.69	6.56	0.82
**Positive feelings about the school environment—KID 9**	7.95	0.78	28.31	0.32
**The ability to concentrate and pay attention—KID 10**	13.56	0.63	33.48	0.24

Note: * Results showing statistical significance.

## Data Availability

The data are available upon reasonable request from the corresponding author.

## Supplementary Materials

The following supporting information can be downloaded at: https://www.mdpi.com/article/10.3390/cancers16213599/s1, Table S1. Percentage distribution of HBSC questionnaire responses obtained in the intervention and control groups, before the intervention and after the intervention. Table S2. Percentage distribution of responses obtained on the KIDSCREEN 10 questionnaire in the intervention and control groups, before the intervention and after the intervention. Table S3. Correlation of the level of physical activity of children in the intervention group with the level of quality of life. Table S4. Correlation of the level of physical activity of children in the control group with the level of quality of life.
