# Supplementary material for: The Impact of Interactive Video Games Training on the Quality of Life of Children Treated for Leukemia
Source: Cancers (Basel). 2024 Oct 25;16(21):3599. doi: 10.3390/cancers16213599 (PMC11545673; doi:10.3390/cancers16213599)
Supplement: Supplementary file 1 [file cancers-16-03599-s001.zip › cancers-3266447-supplementary.pdf]

Table S1. Percentage distribution of HBSC questionnaire responses obtained in the intervention and control groups, before the intervention and after the intervention.

	Intervention group before IVGs program								Intervention group after IVGs program							
**Answers [%]***	HBSC 1.1*	HBSC 1.2*	HBSC 1.3*	HBSC 1.4*	HBSC 1.5*	HBSC 1.6*	HBSC 1.7*	HBSC 1.8*	HBSC 2.1*	HBSC 2.2*	HBSC 2.3*	HBSC 2.4*	HBSC 2.5*	HBSC 2.6*	HBSC 2.7*	HBSC 2.8*
**0**	90.00	0.00	0.00	0.00	0.00	0.00	0.00	0.00	0.00	0.00	0.00	0.00	0.00	0.00	0.00	0.00
**1**	10.00	0.00	0.00	0.00	0.00	0.00	0.00	0.00	0.00	0.00	0.00	0.00	0.00	0.00	0.00	0.00
**2**	0.00	0.00	0.00	0.00	0.00	0.00	0.00	0.00	20.00	80.00	0.00	0.00	0.00	0.00	10.00	0.00
**3**	0.00	0.00	0.00	0.00	0.00	0.00	0.00	0.00	80.00	20.00	10.00	10.00	10.00	10.00	0.00	10.00
**4**	0.00	0.00	0.00	0.00	10.00	10.00	0.00	0.00	0.00	0.00	50.00	0.00	50.00	40.00	20.00	20.00
**5**	0.00	10.00	30.00	10.00	50.00	20.00	40.00	10.00	0.00	0.00	40.00	60.00	40.00	40.00	40.00	60.00
**6**	0.00	90.00	40.00	60.00	40.00	40.00	60.0	30.00	0.00	0.00	0.00	20.00	0.00	10.00	30.00	10.00
**7**	0.00	-	30.00	30.00	0.00	30.00	0.00	60.00	0.00	-	0.00	10.00	0.00	0.00	0.00	0.00
**8**	-	-	0.00	0.00	0.00	0.00	0.00	0.00	-	-	0.00	0.00	0.00	0.00	0.00	0.00
	**Control group at the beginning of the study period**								**Control group at the end of the study period**							
**Answers [%]***	HBSC 1.1*	HBSC 1.2*	HBSC 1.3*	HBSC 1.4*	HBSC 1.5*	HBSC 1.6*	HBSC 1.7*	HBSC 1.8*	HBSC 2.1*	HBSC 2.2*	HBSC 2.3*	HBSC 2.4*	HBSC 2.5*	HBSC 2.6*	HBSC 2.7*	HBSC 2.8*
**0**	90.91	0.00	0.00	0.00	0.00	0.00	0.00	0.00	90.91	0.00	0.00	0.00	0.00	0.00	0.00	0.00
**1**	9.09	0.00	0.00	0.00	0.00	0.00	0.00	0.00	9.09	0.00	0.00	0.00	0.00	0.00	0.00	0.00
**2**	0.00	0.00	0.00	0.00	0.00	0.00	0.00	0.00	0.00	0.00	0.00	0.00	0.00	0.00	0.00	0.00
**3**	0.00	0.00	0.00	0.00	0.00	0.00	0.00	0.00	0.00	0.00	0.00	0.00	0.00	0.00	0.00	0.00
**4**	0.00	0.00	9.09	0.00	9.09	9.09	0.00	0.00	0.00	0.00	9.09	0.00	9.09	0.00	0.00	0.00
**5**	0.00	9.09	45.45	36.36	27.27	9.09	36.36	18.18	0.00	9.09	45.45	27.27	27.27	9.09	36.36	18.18
**6**	0.00	90.91	27.27	27.27	63.64	63.64	63.64	45.45	0.00	90.91	27.27	36.36	63.64	72.73	63.64	45.45
**7**	0.00	-	18.18	36.36	0.00	18.18	0.00	36.36	0.00	-	18.18	36.36	0.00	18.18	0.00	36.36
**8**	-	-	0.00	0.00	0.00	0.00	0.00	0.00	-	-	0.00	0.00	0.00	0.00	0.00	0.00

Note: * The responses and questions were included in a previous paper [39].

Table S2. Percentage distribution of responses obtained on the KIDSCREEN 10 questionnaire in the intervention and control groups, before the intervention and after the intervention.

	Intervention group before IVGs program										Grupa interwencyjna po programie IVGs									
**Answers [%]**	KID 1.1*	KID 1.2*	KID 1.3*	KID 1.4*	KID 1.5*	KID 1.6*	KID 1.7*	KID 1.8*	KID 1.9*	KID 1.10*	KID 2.1*	KID 2.2*	KID 2.3*	KID 2.4*	KID 2.5*	KID 2.6*	KID 2.7*	KID 2.8*	KID 2.9*	KID 2.10*
**0**	90.00	80.00	0.00	0.00	10.00	10.00	0.00	70.00	60.00	30.00	0.00	0.00	0.00	0.00	0.00	0.00	0.00	70.00	30.00	10.00
**1**	10.00	20.00	0.00	0.00	30.00	60.00	0.00	20.00	40.00	60.00	0.00	0.00	80.00	70.00	40.00	0.00	0.00	30.00	60.00	60.00
**2**	0.00	0.00	0.00	60.00	60.00	10.00	0.00	10.00	0.00	10.00	20.00	30.00	20.00	30.00	40.00	40.00	0.00	0.00	10.00	30.00
**3**	0.00	0.00	60.00	30.00	0.00	20.00	10.00	0.00	0.00	0.00	80.00	70.00	0.00	0.00	20.00	60.00	10.00	0.00	0.00	0.00
**4**	0.00	0.00	40.00	10.00	0.00	0.00	90.00	0.00	0.00	0.00	0.00	0.00	0.00	0.00	0.00	0.00	90.00	0.00	0.00	0.00
	**Control group at the beginning of the study period**										**Control group after the research period**									
**Answers [%]**	KID 1.1*	KID 1.2*	KID 1.3*	KID 1.4*	KID 1.5*	KID 1.6*	KID 1.7*	KID 1.8*	KID 1.9*	KID 1.10*	KID 2.1*	KID 2.2*	KID 2.3*	KID 2.4*	KID 2.5*	KID 2.6*	KID 2.7*	KID 2.8*	KID 2.9*	KID 2.10*
**0**	81.82	63.64	0.00	0.00	0.00	27.27	0.00	81.82	72.73	45.45	81.82	81.82	0.00	0.00	0.00	27.27	0.00	81.82	72.73	45.45
**1**	18.18	36.36	0.00	0.00	36.36	36.36	0.00	18.18	27.27	54.55	18.18	18.18	0.00	0.00	36.36	36.36	0.00	18.18	27.27	54.55
**2**	0.00	0.00	0.00	27.27	63.64	36.36	0.00	0.00	0.00	0.00	0.00	0.00	0.00	18.18	63.64	36.36	0.00	0.00	0.00	0.00
**3**	0.00	0.00	63.64	72.73	0.00	0.00	18.18	0.00	0.00	0.00	0.00	0.00	72.73	81.82	0.00	0.00	18.18	0.00	0.00	0.00
**4**	0.00	0.00	36.36	0.00	0.00	0.00	81.82	0.00	0.00	0.00	0.00	0.00	27.27	0.00	0.00	0.00	81.82	0.00	0.00	0.00

Note: * The responses and questions were included in a previous paper [39].

Table S3. Correlation of the level of physical activity of children in the intervention group with the level of quality of life.

Spearman's rank correlation	**Physical activity at the level of MVPA [number of days per week]**		**Vigorous intensity exercise [days per week]**		**TV/movies per week [h]**		**TV/movies on days off [h]**		**NA[a] games per week [h]**		**NA[a] games on days off [h]**		**Use of electronic devices etc. per week [h]**		**Using electronic devices on days off[h]**	
	p-value	Spearman's Rho	p-value	Spearman's Rho	p-value	Spearman's Rho	p-value	Spearman's Rho	p-value	Spearman's Rho	p-value	Spearman's Rho	p-value	Spearman's Rho	p-value	Spearman's Rho
**Well-being and physical fitness - KID 1**	0.38	0.37	1	0.25	1	-0,14	0.93	0.2	0.24	-0,58	1	0	0.87	-0.05	0.93	0.2
**Feeling strong and energetic - KID 2**	1	0.22	1	-0.22	**0.03***	-0.76	0.69	-0.21	1	0	0.43	-0.36	0.52	-0.28	0.47	-0.26
**Feeling of sadness - KID 3**	1	0.25	1	-0.25	0.24	0.58	0.93	-0.2	1	0.14	1	0	0.11	0.64	0.93	0.2
**Feeling of loneliness - KID 4**	1	-0.22	0.53	-0.33	1	0	1	0.08	0.45	0.38	0.9	-0.16	0.67	-0.2	0.28	-0.43
**Having time for oneself - KID 5**	0.15	0.56	0.15	-0.6	0.74	0.13	0.16	0.53	0.42	0.32	0.14	0.53	0.11	0.55	0.11	0.56
**Engaging in activities one felt like doing - KID 6**	1	0.10	1	-0.10	0.38	0.35	0.56	0.28	0.38	-0.35	0.71	0.2	0.99	0.04	0.27	0.44
**Good relations with parents - KID 7**	1	-0.17	1	0.17	0.5	-0.38	1	0.13	1	0.19	1	0.25	0.4	-0.43	1	-0.13
**Good relationship with peers, fun - KID 8**	1	-0.22	0.53	-0.33	1	0	1	0.09	1	0	0.17	-0.49	0.67	-0.2	0.28	-0.43
**Positive feelings about school environment - KID 9**	0.07	0.7	1	0.1	0.46	-0.24	0.93	-0.03	0.46	-0.24	0.36	-0.37	0.99	-0.03	0.21	-0.42
**The ability to focus and pay attention - KID 10**	0.2	0.55	1	-0.1	1	-0.06	0.96	-0.02	0.83	-0.13	0.59	-0.24	0.83	-0.06	0.53	-0.23

Note: * Results showing correlation; [a] NA – non active video games.

Table S4. Correlation of the level of physical activity of children in the control group with the level of quality of life.

Spearman's rank correlation	**Physical activity at the level of MVPA [number of days per week]**		**Vigorous intensity exercise [days per week]**		**TV/movies per week [h]**		**TV/movies on days off [h]**		**NA[a] games per week [h]**		**NA[a] games on days off [h]**		**Use of electronic devices etc. per week [h]**		**Using electronic devices on days off[h]**	
	p-value	Spearman's	p-value	Spearman's	p-value	Spearman's	p-value	Spearman's	p-value	Spearman's	p-value	Spearman's	p-value	Spearman's	p-value	Spearman's
**Well-being and physical fitness - KID 1**	0.18	0.67	1	0.15	1	0	0.16	0.55	0.62	-0.26	0.16	-0.52	0.49	0.36	0.31	-0.44
**Feeling strong and energetic - KID 2**	0.18	0.67	1	0.15	1	0	0.67	0.24	1	-0.09	0.96	-0.09	0.49	0.36	1	-0.08
**Feeling of sadness - KID 3**	1	-0.19	0.27	-0.52	0.48	0.31	1	-0.07	1	0.08	0.56	0.29	1	0.04	0.36	0.42
**Feeling of loneliness - KID 4**	1	0.15	1	-0.15	**0.02***	-0.71	0.16	-0.55	1	0.09	0.45	-0.38	0.49	-0.36	0.67	-0.20
**Presence of time for oneself - KID 5**	1	0.24	1	-0.24	0.39	0.29	0.46	0.32	0.89	0.14	0.83	0.15	0.57	0.21	0.68	0.19
**Engaging in activities one felt like doing - KID 6**	0.64	0.37	1	0.05	0.65	0.16	0.26	0.41	1	0.01	0.38	-0.25	1	0.06	0.76	0.11
**Good relations with parents - KID 7**	1	0.15	1	-0.15	0.69	-0.2	0.67	-0.24	1	0.09	0.96	0.09	1	0.13	0.13	-0.56
**Good relationship with peers, fun - KID 8**	1	-0.15	1	0.15	0.69	0.2	0.67	0.24	1	-0.09	0.96	-0.09	1	-0.13	0.13	0.56
**Positive feelings about school environment - KID 9**	1	-0.19	1	0.19	0.55	-0.17	0.43	-0.31	1	0.08	0.56	0.29	0.49	-0.39	0.17	-0.45
**The ability to focus and pay attention - KID 10**	1	0.29	0.45	0.35	0.11	0.55	**0.02***	0.79	0.47	-0.34	0.76	-0.18	0.24	0.45	0.62	0.25

Note: * Results showing correlation; a NA – non active video games.
